# Body Image, Nutrition, and Mental Health

**DOI:** 10.3390/nu16081106

**Published:** 2024-04-10

**Authors:** Hubertus Himmerich, Khadijeh Mirzaei

**Affiliations:** 1 Department of Psychological Medicine, Institute of Psychiatry, Psychology & Neuroscience, King’s College London, London SE5 8AF, UK; 2 Department of Community Nutrition, School of Nutritional Sciences and Dietetics, Tehran University of Medical Sciences (TUMS), Tehran, Iran; mirzaei_kh@sina.tums.ac.ir

Classical examples of disorders associated with body image disturbances are eating disorders (EDs) such as anorexia nervosa (AN) and bulimia nervosa (BN), as well as body dysmorphic disorder (BDD). Body image is a complex construct comprising thoughts, feelings, evaluations, and behaviors related to one’s body. Body image disturbances are not exclusively found in EDs and BDD; they are also highly prevalent in people with other mental or physical health problems, e.g., depression or obesity, and in the general population [1].

Similarly, altered nutritional intake is not restricted to EDs and obesity. A change in food intake can be a symptom of a mental health disorder such as depression [2], or a consequence of psychopharmacological treatment [3]. Increased appetite and food intake lead to obesity, which is often associated with depression [4]. Vice versa, weight loss can help with depression in people with obesity if they lose weight under a calorie-restricted diet [5]. These examples indicate the close association between nutrition and mental health disorders.

This Special Issue examines and illuminates the complex relationships between body image, nutrition, and mental health. More specifically, it covers the psychological and social risk factors of body image disturbances and associated disorders, biological aspects of appetite regulation and the metabolic syndrome, and therapeutic approaches for EDs and weight disorders and their health consequences.

Psychosocial risk factors of body image disturbances and associated disorders are the first group of themes covered in this Special Issue.

Yumen et al. [6] and Karam et al. [7] examined the influence of social websites and social media use on body image and food intake. In a sample of young Japanese women, Yumen et al. found that longer social networking site use was associated with lower body weight and with a thinner body shape ideal [6]. Karam et al. researched the relationship between social media use and body image in Lebanese university students. Individuals with more social media use had higher odds of exhibiting body image concerns and were at risk for emotional overeating [7].

Witaszek et al. investigated anxiety and depression as risk factors of the use of food intake to regulate emotions and found that women with anxiety and depression showed higher scores for uncontrolled and emotional eating but lower scores of cognitive restraint [8]. As an additional result, they reported that the use of the glucagon-like peptide 1 (GLP-1) receptor agonists liraglutide and semaglutide was associated with increased cognitive restraint in the Three-Factor Eating Questionnaire.

In a study using a virtual reality (VR) kitchen as experimental paradigm, Bektas et al. observed that ED symptoms correlated positively with food-specific trait and state disgust [9].

Baceviciene et al. compared disordered eating, body image, and sociocultural and coach-related pressures between adolescent and adult and between male and female athletes. An interesting finding was that vomiting, laxative misuse, and excessive exercise were more prevalent in adolescent female athletes than adult females, while dietary restraint was more common in adult male athletes compared to adolescent males. Adolescent female athletes experienced a high sociocultural pressure from family and peers, and sport-related pressure from the coach [10].

Fabrig et al. examined a large sample of candidates for bariatric surgery and found a strong association between weight-related experienced stigmatization and depressive symptoms in candidates with high weight bias internalization [11].

Biological appetite regulation and markers of the metabolic syndrome constitute the second group of themes covered in this Special Issue.

In a sophisticated immunohistochemical study, Fichtner et al. provided evidence for a widespread distribution of the glial-cell-derived neurotrophic factor (GDNF) receptor alpha-like (GFRAL), which is the receptor for growth differentiation factor-15 (GDF-15). They found GFRAL in the prefrontal cortex, the hippocampus, the arcuate nucleus, and in peripheral tissues. GFRAL had already been implicated in food intake and body weight regulation, but these novel findings indicate a broader role of GFRAL in metabolism [12].

Bilska et al. investigated markers of metabolic syndrome and adipokines in a sample of patients with a depressive episode of bipolar disorder. They found that in women with bipolar depression, visfatin, S100B, and leptin concentrations correlated with metabolic syndrome, whereas adiponectin and leptin-receptor levels were negatively associated with it [13].

Therapeutic approaches for EDs and weight disorders and their health consequences are the third group of themes covered in this Special Issue.

Clemente-Suárez et al. summarized the non-pharmacological interventions in people with AN in a narrative review and thus covered nutritional interventions, psychological and family therapy, social media use management, and physical therapy interventions including relaxation, massages, and exercises [14].

The therapeutic use of the novel technology-enabled smart toy Purrble, which is designed for emotional regulation, was explored in a mixed-method analysis by Chubinidze et al. [15]. They found that it might be helpful particularly for patients with EDs and complex presentations.

A systematic review and meta-analysis of randomized controlled trials to treat obesity in military populations was performed by Gravina et al. [16]. They found that the current weight loss interventions are effective in military populations with a high level of evidence for physical activity, dietary and nutritional interventions, cognitive behavioral therapy, and structured outcome monitoring (clinical or self-monitoring).

Paszynska et al. summarized the biological, behavioral (binge eating episodes, vomiting, acidic diet, poor oral hygiene), and pharmacotherapeutic factors that may threaten oral health in people with EDs [17]. In their article, they advocate for early diagnosis, reductions in behaviors that are destructive for oral health, nutritional counselling, and medical interventions to treat and protect oral soft and hard tissues.

In summary, the use of social websites and social media, anxiety and depression, food-specific disgust, social pressure, physical fitness pressure, weight-related experienced stigmatization, and weight bias internalization were identified as risk factors for body image disturbances; disordered, restrictive, or over-eating; vomiting; laxative misuse; excessive exercise; and associated mental health problems [7,8,9,10,11]. The GDF-15 receptor GFRAL, which is known to be involved in appetite regulation, was found to be expressed across various central-nervous as well as peripheral tissues [12], and visfatin, S100B, and leptin are associated with the development of metabolic syndrome in people with bipolar depression [13]. Four articles of this Special Issue [14,15,16,17] summarized already available (e.g., nutritional interventions, psychological and family therapy) [14] and experimental (smart toy Purrble) [15] therapies for AN, effective therapies for the treatment of obesity (e.g., physical activity, dietary and nutritional intervention, cognitive behavioral therapy, clinical and self-monitoring) [16], as well as the preventive and therapeutic options for oral health consequences of EDs [17]. Witaszek et al.’s finding that liraglutide and semaglutide were associated with increased cognitive restraint [8] is also therapeutically relevant as these GLP-1 receptor agonists are approved for the treatment of obesity.

The mentioned findings reported in this Special Issue are clinically relevant and pave the way for future research in EDs and weight-related disorders.

For example, as weight gain is a clinically significant problem during the treatment with antidepressants and antipsychotics [3], prescription of the GLP-1 receptor agonists liraglutide and semaglutide alongside the treatment with weight-gain-inducing psychopharmacological medications might help to prevent or attenuate weight gain [18]. Witaszek et al.’s finding that liraglutide and semaglutide were associated with increased cognitive restraint [8] might help to explain weight loss during treatment with these GLP-1 receptor agonists.

The findings of Bilska et al. [13] that visfatin, S100B, and leptin concentrations correlated with the metabolic syndrome point to the involvement of the immune system in EDs and weight disorders, because visfatin, S100B, and leptin modulate the release of pro-inflammatory cytokines like tumor necrosis factor (TNF)-α and interleukin (IL)-6 [19,20,21]. TNF-α and IL-6 have been found elevated in people with obesity [22] but also in patients with AN [23]. Moreover, TNF-α and IL-6 inhibitors have been reported to lead to an increase in body weight and were therefore suggested as potential future treatments of AN [24,25]. Taking their influence on mood, cognition and behavior into account, these cytokines might not only be involved in the pathophysiology of EDs but also other mental health disorders [26]. Vice versa, psychopharmacological agents that treat mental health disorders like antipsychotics, antidepressants, and mood stabilizers have been shown to alter cytokine production in vivo and in vitro [27,28].

By using novel technical solutions such as VR [9] or a smart toy [15] as add-ons, therapists might enhance the success of their therapies in the future.

To conclude, this Special Issue covers psychological, social, and biological aspects of mental health problems associated with body image disturbances, and over- and undernutrition. It also provides novel ideas for psychometric and biological markers and therapeutic options for people with EDs and weight disorders such as the use of GLP-1 receptor agonists, GDF-15 and GFRAL signaling modification, and the enhancement of psychotherapies using VR and smart toys.
